# Comparison of clinical outcomes between Castor single-branched stent graft and in situ fenestration in treating Stanford type B aortic dissection involving the left subclavian artery: A retrospective case-control study

**DOI:** 10.1097/MD.0000000000040623

**Published:** 2024-11-22

**Authors:** Xiang Weng, Teng Zhang, YiLiang Hu, XianGui Li, Weimin Zhou

**Affiliations:** a Department of Vascular Surgery, the Second Affiliated Hospital, Jiangxi Medical College, Nanchang University.

**Keywords:** Castor stent, in situ fenestration technique, left subclavian artery, thoracic endovascular aortic repair of aortic dissection

## Abstract

Stanford type B aortic dissection involving the left subclavian artery (LSA) poses significant clinical challenges. The Castor single-branch stent graft and in situ fenestration are commonly used techniques, but the better endovascular treatment remains debated. This study evaluates the clinical effects of the Castor single-branched stent graft versus in situ fenestration in treating Stanford type B aortic dissection involving the LSA. We selected 75 patients with Stanford type B aortic dissection involving the LSA, admitted to the Second Affiliated Hospital of Nanchang University from January 2018 to May 2022. All patients underwent thoracic endovascular aortic repair; 34 received the Castor single-branched stent graft, while 41 underwent in situ fenestration. Clinical efficacy and perioperative complications were compared. The technical success rate of the Castor single-branch stent graft was significantly higher than that of in situ fenestration (97.06% vs 80.49%, *P* = .04). The Castor group exhibited significantly better outcomes in operative time (136.45 ± 25.53 min vs 157.08 ± 18.14 min), LSA blood flow recovery time (6.8 ± 2.3 min vs 20.1 ± 9.8 min), blood loss (29.03 ± 9.78 mL vs 35.69 ± 10.77 mL), contrast medium usage (288.71 ± 72.70 mL vs 352.78 ± 81.02 mL), and immediate postoperative endoleaks (1/34 vs 7/41) (*P* < .05). Stroke incidence (1/34 vs 2/41) and perioperative mortality (0/34 vs 1/41) were similar (*P* > .05). Hospital stays were comparable (15.71 ± 6.04 days vs 14.22 ± 5.01 days, *P* = .28). However, the cost of medical supplies (154,168.62 ± 28,288.44 CNY vs 119,589.72 ± 34,199.67 CNY) and total hospital expenses (192,665.88 ± 40,027.99 CNY vs 153,920.47 ± 42,670.87 CNY) were significantly higher in the Castor group (*P* < .05). The median follow-up time was 9 months (1–60 months). Follow-up showed similar 30-day postoperative mortality (1/41 vs 0/34, *P* = 1.00), stent patency (33/34 vs 39/41, *P* = .67), and stent migration rates (0/34 vs 1/41, *P* = 1.00). However, the endoleak rate was significantly higher in the in situ fenestration group (11/41 vs 1/34, *P* = .01). Both the Castor single-branch stent graft and the in situ fenestration technique effectively protect and reconstruct the LSA in Stanford type B aortic dissection, with the Castor single-branch stent graft showing higher technical success and fewer complications, indicating better clinical potential.

## 1. Introduction

Thoracic aortic dissection is 1 of the most dangerous diseases affecting human health. If it is not treated in time, the mortality rate of patients is 22.7% within 6 hours, up to 50% within 24 hours, and the mortality rate will rise to 68% within 1 week^.[1]^ Thoracic aortic endovascular repair (TEVAR) is an important treatment for thoracic aortic dissection.^[2,3]^ The purpose of TEVAR is to close the proximal tear with a covered stent graft, thereby inducing thrombosis of the false lumen, expansion of the true lumen, and restoration of blood flow. In patients with Standford type B aortic dissection (TBAD), the primary tear usually arises from the left subclavian artery (LSA), a few centimeters distal to the entrance.^[4,5]^ Mesar et al found that 89.5% of TBAD patients had a healthy proximal descending aorta of <2 cm, implying a lack of an adequate proximal anchoring zone.^[6]^ A clear definition of an adequate proximal anchoring area means that the area between the proximal edge of the stented graft and the proximal tear is adequately covered and firmly secured when performing a stented graft procedure, a step that is critical to the success of the procedure. Thus, proximal extension of the stented graft is necessary when an adequate proximal anchoring zone is lacking. It has been reported that LSA coverage is 1 way to create a sufficient anchoring zone; However, as found in clinical practice, LSA coverage is associated with increased risk of stroke and spinal cord ischemia.^[7,8]^ Therefore, it is crucial to better preserve and reconstruct the LSA to reduce the risk of stroke and spinal cord ischemia. Although traditional open surgery can effectively preserve the branch vessels of the superior arch, it also has the disadvantages of high invasiveness, reliance on extracorporeal circulation, deep hypothermic circulatory arrest, and relatively large surgical complications.^[5]^ The continuous development of minimally invasive techniques and materials has led to the emergence of a variety of advanced vascular intervention techniques, including parallel (chimney) stents, “submerged mirror” techniques, fenestration, and branch stents, etc, which can effectively expand the proximal landing zone.^[6]^ Among them, fenestration techniques include in situ fenestration technique and in vitro pre-fenestration technique, which are commonly used surgical approaches to preserve supra-arch branch vessels, and their operation difficulty and operation risk are significantly lower than those of open surgery.^[7]^ Castor single-branched stent transplantation also has lower operation difficulty and surgical risk and can maintain the blood supply of LSA.^[9]^ The aim of this study is to compare and evaluate the clinical efficacy of Castor single-branched stent graft and in situ fenestration technique in the treatment of type B aortic dissection involving the LSA, and to provide a reference for the treatment of TBAD involving the LSA.

## 2. Materials and methods

### 2.1. General information

The clinical data of patients with Standford type B aortic dissection involving the LSA who underwent surgical treatment in the Second Affiliated Hospital of Nanchang University from January 2018 to May 2022 were retrospectively analyzed. Inclusion criteria: Standford type B aortic dissection was diagnosed by computed tomography angiography (CTA) or angiography and treated by interventional surgery; patients had complete preoperative CTA image data, and at least 1 follow-up CT angiography (CTA) or angiography within 6 months after surgery. Exclusion criteria: surgical treatment with chimney or external pre-fenestration techniques; patients with active Marfan syndrome, syphilis or aortitis; loss of follow-up or incomplete clinical data. A total of 212 patients with TBAD who underwent TEVAR in our center from January 2018 to May 2023 were enrolled. Among them, 113 patients were reconstructed with LSA due to insufficient anchor point, 12 patients who were reconstructed with chimney technique and 5 patients who were reconstructed with external PR fenestration technique were excluded, and 21 patients were lost to follow-up. A total of 75 patients were enrolled, of which 34 patients were treated with Castor single-branched stent graft and 41 patients within situ fenestration technique. There were no significant differences in age, gender, timing of treatment (elective surgery and emergency surgery), comorbidities and aortic arch classification between the 2 groups *P* > .05 (Table 1). This study was approved and monitored by the Biomedical Research Ethics Committee of the Second Affiliated Hospital of Nanchang University.

**Table 1:** Baseline clinical data of patients with type B aortic dissection involving LSA.

	Castor stent graft (n = 34)	In situ fenestration technique (n = 41)	*P*-value
Age (yr, mean ± SD)	52.9 ± 13.1	60.2 ± 10.9	.276
Sex			
Male	30 (88.2%)	35 (85.4%)	.382
Female	4 (11.8%)	6 (14.6%)	
Treatment timing			
Selective operation	16 (47.1%)	21 (51.2%)	.521
Emergency operation	18 (52.9%)	20 (48.8%)	
Combined disease			
Hypertension	24 (70.6%)	32 (78.0%)	.596
Diabetes	2 (5.9%)	4 (9.8%)	
Coronary heart disease	1 (2.9%)	0 (0.0%)	
Hyperlipemia	2 (5.9%)	2 (4.9%)	
Renal insufficiency	3 (8.8%)	4 (9.8%)	
Atrial fibrillation	3 (8.8%)	0 (0.0%)	
Cerebral infarction	0 (0.0%)	4 (9.8%)	
Arch type			
I	13 (38.2%)	14 (34.1%)	.579
II	10 (29.4%)	15 (36.6%)	
III	11 (32.4%)	12 (29.3%)	

*P* < .05 was considered as statistically significant; LSA = left subclavian artery.

### 2.2. Preoperative preparation

After the patients were diagnosed with Sandford type B aortic dissection by CTA or angiography before operation, the type of aortic arch, the distance from the tear to the distal end of LSA, the length of the normal landing zone, and the distance between the 3 branches of the aortic arch were fully evaluated. According to the evaluation results, the surgical method was determined, and the reasonable choice of stents was optimized. All patients completed detailed preoperative examination, excluded surgical contraindications, and signed the informed consent for surgery.

### 2.3. Surgical procedures

#### 2.3.1. In situ single fenestration technique

**Figure 1.**
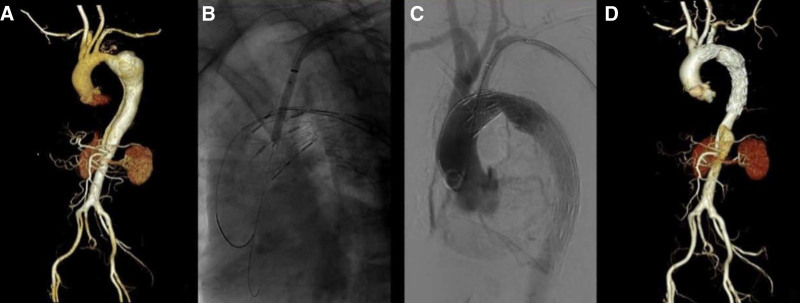
(A) CTA showed that the proximal tear of the aortic dissection was located at the greater curvature of the descending aorta, and the dissection involved the distal end of the LSA. (B, C) Intraoperative angiography showed that the stent was released accurately, the 3 branches of the arch were well developed, the tear was completely closed, the false lumen disappeared, and there was no internal fistula. (D) Follow-up CTA showed that the stent was in good position without internal fistula and the 3 branches above the arch were well developed. CTA = computed tomography angiography, LSA = left subclavian artery.

The patient was placed in a supine position and the bilateral inguinal region and left upper limb were antisepticised. After local anesthesia (anesthesia followed by disinfection during general anesthesia), the right common femoral artery was punctid, 2 ProGlide vascular occluders were preset, and a 14F vascular sheath was inserted. At the same time, the skin was cut about 5cm along the brachial artery medial to the left elbow, and the left brachial artery was separated and exposed for use. The gold labeled catheter was sent to the ascending aorta under the guidance of the super smooth guide wire through the right femoral artery path, and the lesion was determined by angiography and measured. According to the measurement results and the results of preoperative imaging examination, the best type of thoracic aortic covered stent was selected, and the thoracic main covered stent was sent to the ascending aorta under the guidance of the Lunderquist super hard guide wire. It was located at the posterior edge of the LSA under Digital Subtraction Angiography (DSA) fluoroscopy and released after accurate positioning. A 2.6F-90cm polished V18 guide wire and support catheter were inserted into the sheath. The membrane was ruptured by puncture with the catheter and guide wire. After successful rupture, 0.018 “system 2.5 to 4.0 mm small balloons were used for predilation in sequence. Then, the 0.035 “system 4 to 12 mm large balloon was replaced to gradually expand the tear until satisfactory, and the best type of vascular covered stent was introduced, which was accurately released at the beginning of the LSA determined by DSA fluoroscopy. After the large balloon was used to expand again, the stent delivery device was withdrawn. Re-angiography showed that the stent was accurately positioned, the tear was completely covered, the 3 branches of the aortic arch were well developed, and there was no endoleak between the stent and the aorta (Fig. 1).

#### 2.3.2. Castor single-branched stent graft

**Figure 2.**
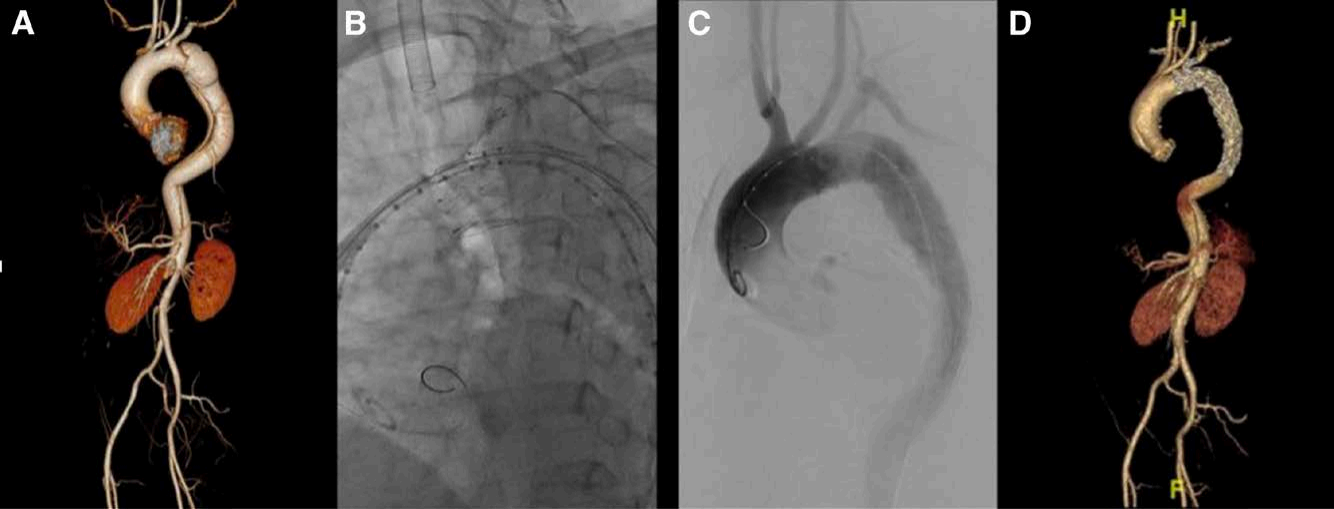
(A) CTA showed the dissection was close to the LSA and involved the thoracoabdominal aorta. (B, C) angiography showed satisfactory stent release without internal fistula. (D) Follow-up CTA showed that the stent was in good position without internal fistula and the branches were well developed. CTA = computed tomography angiography, LSA = left subclavian artery.

The patient was placed in the supine position, and the bilateral inguinal area and left upper limb were sterilized. Two ProGlide vascular occlusive devices and a 5F vascular sheath were prepositioned in the right femoral artery, and a 7F vascular sheath was placed in the left brachial artery. A gold labeled catheter was placed into the ascending aorta under the guidance of a super smooth guide wire through the right femoral artery approach. After angiography, the lesion was identified and measured, and the best type of Castor single-branched stent was selected according to the results of preoperative imaging examination. Subsequently, an ultra-smooth guide wire with a multifunctional catheter was routed through the left brachial artery, LSA, and descending aorta to the right femoral artery. The catheter was delivered into the 5F vascular sheath in conjunction with a guide wire, and the multifunctional catheter was subsequently removed from the body. Under the guidance of Lunderquist super-hard guide wire, the stent was placed into the descending aorta through the right femoral artery, and the branch segment traction wire was pulled out from the sheath of the left brachial artery. Then the stent was delivered to the planned position in the aortic arch. The branch stent was pulled into the LSA by the traction wire, and the main stem of the stent was released. Re-angiography showed that the stent was accurately positioned, the tear was completely covered, the 3 branches of the aortic arch were well developed, and there was no endoleak between the stent and the aorta (Fig. 2).

### 2.4. Observation and follow-up indicators

The perioperative related indicators of the 2 groups were statistically analyzed, including technical success rate (the surgery was successfully completed, with the patient’s vital signs stable. Angiography showed smooth blood flow within the stent, good sealing of the proximal tear, no significant endoleak, and no stenosis in the LSA stent), operation time, LSA blood flow recovery time, blood loss, and contrast agent dosage. The length of hospital stays and treatment cost of patients in the 2 groups were recorded. Procedural complications including immediate endoleak after stent release, cerebral infarction and perioperative death were recorded. Postoperative follow-up: the survival rate and secondary surgical intervention during the follow-up period were recorded. Postoperative follow-up with chest aorta CTA will be conducted at 1, 3, and 6 months, 1 year, and annually thereafter to evaluate whether the stent has dislocated, assess its morphology, check for endoleaks, and determine if there is any stenosis or occlusion in the branch stents. The follow-up time for this study was the time corresponding to the patient’s most recent CTA findings.

### 2.5. Statistical analysis

Descriptive statistics were used, with count data presented as n (%), and measurement data expressed as mean ± standard deviation (x̅ ± s). An independent samples *t*-test was applied for the comparison of measurement data between 2 groups, and the chi-square test was used for categorical data (such as frequency data). If *P* < .05, the difference was considered statistically significant.

## 3. Results

### 3.1. Outcomes

The primary outcome was the technical success rate of the procedures, defined as the successful deployment of the stent or fenestration with adequate blood flow restoration to the LSA. Secondary outcomes included operation time, blood loss, contrast agent usage, recovery time of LSA blood flow, incidence of postoperative endoleak, and overall complications, including cerebral infarction and perioperative mortality.

### 3.2. Results of surgery

Both surgical approaches demonstrated high technical success rates, with the Castor single-branch stent graft group achieving a slightly higher success rate than the in situ fenestration group, a difference that was statistically significant (*P* = .04). The technical success rate of the in situ fenestration group was 80.49% (33/41), with 1 case of intraoperative death due to pericardial tamponade caused by retrograde dissection, and 7 cases of intraoperative stent endoleak. The success rate in the Castor group was 97.06% (33/34), with only 1 case of intraoperative endoleak. The Castor group had significantly better outcomes in terms of operative time (136.45 ± 25.53 min vs 157.08 ± 18.14 min), LSA blood flow recovery time (6.8 ± 2.3 min vs 20.1 ± 9.8 min), blood loss (29.03 ± 9.78 mL vs 35.69 ± 10.77 mL), contrast medium usage (288.71 ± 72.70 mL vs 352.78 ± 81.02 mL), and the incidence of immediate postoperative endoleak (1/34 vs 7/41) (*P* < .05). The incidence of stroke (1/34 vs 2/41) and perioperative mortality (0/34 vs 1/41) were similar between the 2 groups, with no statistically significant differences (*P* > .05) (Table 2). There was no significant difference in hospital stay between the 2 groups (15.71 ± 6.04 days vs 14.22 ± 5.01 days, *P* = .28), but the Castor group had significantly higher costs for medical supplies (154,168.62 ± 28,288.44 CNY vs 119,589.72 ± 34,199.67 CNY) and total hospitalization expenses (192,665.88 ± 40,027.99 CNY vs 153,920.47 ± 42,670.87 CNY) (*P* < .05) (Table 3).

**Table 2:** Results of perioperative related indicators.

Indicators	Castor stent graft (n = 34)	In situ fenestration technique (n = 41)	*t*/χ^2^	*P*
Technical success rate	33/34	33/41	3.40	.04
Operation time (min)	142.53 ± 24.64	160.80 ± 19.65	3.857	.00
LSA blood flow recovery time (min)	9.79 ± 4.87	22.39 ± 7.05	9.12	.00
Blood loss (mL)	29.12 ± 9.41	35.00 ± 10.78	2.49	.02
Contrast agent dosage (mL)	289.71 ± 73.63	331.59 ± 112.41	1.87	.00
Endoleak occurred immediately after stent release	1/34	7/41	3.90	.048
Cerebral infarction	1/34	2/41	0.00	1.00
Death occurred during perioperative period	0/34	1/41	0.00	1.00

*P* < .05 was considered as statistically significant; LSA = left subclavian artery.

**Table 3:** Comparison of length of stay and related treatment costs between the 2 groups.

Group of groups	Length of hospital stay (d)	Total cost (Yuan)	Cost of consumables (Yuan)
Castor stent graft (n = 34)	15.79 ± 5.89	190,655.41 ± 38,727.19	153,403.18 ± 27,096.24
In situ fenestration technique (n = 41)	15.51 ± 7.16	157,121.39 ± 43,011.74	121,533.90 ± 34,921.95
*t*	0.18	3.52	4.45
*P*	.86	.00	.00

*P* < .05 was considered as statistically significant.

### 3.3. Results of follow-up

By March 2023, a total of 21 patients were lost to follow-up. The follow-up methods included outpatient visits, WeChat, and telephone communication, with a follow-up duration ranging from 1 to 60 months. The median follow-up time was 9 months, and the mean follow-up time was 12.9 ± 1.0 months. During the follow-up period, 1 patient in the in situ fenestration group experienced proximal stent retrograde tearing 21 days postoperatively, resulting in acute pericardial tamponade and death, leading to a 30-day mortality rate of 1/41. In the Castor single-branch stent graft group, no patients died within 30 days postoperatively, resulting in a 30-day mortality rate of 0/34. There was no statistically significant difference in the 30-day mortality rates between the 2 groups (*P* = 1.00). One patient in the Castor group was found to have LSA stent stenosis on CTA 1 month after surgery, while 2 patients in the in situ fenestration group were found to have LSA stent stenosis during follow-ups at 6 and 9 months postoperatively. None of the 3 patients showed significant symptoms of posterior circulation or limb ischemia, and no further intervention was performed. The patency rate of the LSA stent was 33/34 in the Castor group and 39/41 in the in situ fenestration group, with no statistically significant difference between the 2 groups (*P* = .67). During the follow-up period, 1 patient in the in situ fenestration group experienced stent displacement at the 6-month follow-up CTA, while none of the 34 patients in the Castor group had stent displacement or deformation. The stent displacement or deformation rate between the 2 groups was comparable (1/41 vs 0/34, *P* = 1.00). Intraoperative angiography in the in situ fenestration group revealed endoleak in 7 patients. Among them, 3 cases resolved after intraoperative coil embolization, 2 cases resolved on CTA follow-up at 3 and 9 months, and 2 cases showed worsening endoleak at 3 and 6 months postoperatively (with 1 case developing stent deformation due to severe endoleak), which resolved after a second surgery. Additionally, 4 patients showed no intraoperative endoleak but were found to have endoleak during CTA follow-up at 3, 6, and 12 months postoperatively. The endoleak rate in the in situ fenestration group was 11/41. In contrast, intraoperative angiography in the Castor group identified endoleak in 1 patient, which resolved on CTA at the 3-month follow-up, with an endoleak rate of 1/34. The difference in endoleak rates between the 2 groups was statistically significant (*P* = .01) (Table 4).

**Table 4:** Postoperative follow-up indicators.

	Castor stent graft (n = 34)	In situ fenestration technique (n = 41)	χ^2^	*P*
30-d mortality after surgery	0/34	1/41	0.00	1.00
Stent patency rate of subclavian artery	33/34	39/41	0.18	.67
Rate of stent migration or deformation	0/34	1/41	0.00	1.00
Endoleak rate	1/34	11/41	6.21	.01

*P* < .05 was considered as statistically significant.

## 4. Discussion

At present, TEVAR is the main method for the treatment of thoracic aortic dissection and thoracic aortic aneurysm. With the development of minimally invasive techniques and instruments, its treatment indications are more and more extensive. Conventional TEVAR is a reliable surgical method with simple operation, low risk.^[10]^ However, when the normal anchorage zone is <15 mm and the aortic arch is distorted or angulated, routine coverage of LSA will lead to subclavian steal syndrome, which in turn will affect the blood supply to the brain, especially when the left vertebral artery is the dominant artery.^[11]^ When the lesion involves the ascending aorta or the 3 branches of the upper arch, traditional TEVAR still faces major challenges.^[12]^ Therefore, reconstruction and preservation of supra-arch branch vessels is the difficulty and focus of the treatment of thoracic aortic dissection and aneurysm. It has been proposed that preservation of supra-arch branch vessels by open surgery results in better long-term results, but open surgery is highly invasive and has relatively more severe short-term complications.^[13,14]^ Minimally invasive techniques for preserving supra-arch vessels include hybrid surgery, parallel stent techniques (parallel chimney technique and retrograde submerged mirror technique), fenestration techniques (in situ fenestration and in vitro fenestration), and branched stent techniques. Compared with other techniques, branched stent technique and in situ fenestration technique have unique advantages, including less trauma, high technical success rate, low incidence of branch stent stenosis and occlusion, and better retention of original hemodynamic characteristics.^[15,16]^ Further analysis of this study compared the short- and medium-term clinical efficacy of these 2 surgical methods.

In this study, the perioperative operation related indicators, perioperative complications, hospitalization time, treatment cost and follow-up related indicators of Castor branch stent graft and in situ fenestration technique were statistically analyzed and compared. It was found that both surgical methods had a high technical success rate, with the Castor single-branch stent group achieving a success rate of 97.06% and the in situ fenestration technique group achieving a success rate of 80.49%. The feasibility of preserving LSA in the treatment of TBAD with insufficient proximal landing zone is fully affirmed.

In this study, the incidence of endoleak during the operation of in situ fenestration technique was higher than that of Castor single-branched stent transplantation. The immediate incidence of endoleak during the operation of in situ single fenestration technique in this study was 17.10%, which was slightly lower than the 21.6% reported in the previous study.^[17]^ The use of in situ fenestration technique may not well adapt to the aortic arch structure of the patient, resulting in the “beak phenomenon” caused by the stent not fully fit.^[18]^ In the patients who received the Castor single-branch stent implantation, only 1 case of type I endoleak occurred. The Castor single-branch stent is an integrated stent, and the presence of the branch stent limits the stent’s displacement during the release process to some extent, and its proximal end was designed as a multiple small band of the bare segment, which extended the length of the anchoring region and made it adhere to the aortic wall more closely, thereby reducing the occurrence of endoleak.^[19]^ Both techniques have a low incidence of perioperative cerebral infarction, and the incidence is similar. In this study, a total of 3 cases of cerebral infarction occurred during the perioperative period, including 1 case with Castor stent technique and 2 cases within situ single fenestration technique, which were caused by transient cerebral ischemia, target vessel plaque displacement, air embolism, and small fragments displacement during stent expansion, and were relieved after conservative treatment. Therefore, it is essential to perform CTA or vascular ultrasound examination before operation, minimize the time of window opening, operate cautiously, and use brain protection devices when necessary.^[9]^ In this study, in this study, 1 patient experienced proximal stent retrograde tearing intraoperatively, and another experienced it 21 days postoperatively, both resulting in acute cardiac tamponade and death. Retrograde tear is 1 of the serious complications of TEVAR.^[20]^ Before the operation, it is necessary to evaluate the shape of the aortic arch, the distance between the 3 branch vessels above the arch, the distortion of the aortic arch, and the calcification of the branch vessels, to optimize the selection of stents. At the same time, compared with Castor single-branch stent grafting, the in situ single fenestration technique uses polished sharp V18 guide wire to puncture the stent membrane, which has the risk of bleeding caused by guide wire puncture blood vessels. It is necessary to determine the puncture direction and depth from multiple angles and levels, combine with Fustar adjustable sheath, combine with support catheter, avoid vascular injury and other complications, improve the technical success rate and reduce the amount of intraoperative blood loss. Castor single-branched stent implantation can achieve rapid release of branches in a short time, restore the blood flow of LSA, and without puncture rupture, which can effectively avoid complications such as cerebral infarction and hemorrhage. In situ fenestration and branched stent grafting are widely used in clinical practice because they can maintain the original hemodynamic characteristics, have the advantages of less trauma, low cost, and relatively low incidence of complications.^[21]^ However, when the angle between the branch vessel and the aortic arch is <30°,^[22]^ it is relatively difficult to puncture the stent membrane within situ fenestration technique. A thorough evaluation of the patient by an experienced physician is required. For patients undergoing in situ fenestration, preoperative aortic CTA and carotid ultrasound should be completed to fully evaluate the stage and morphology of the aortic arch, the degree of branch vessel involvement, and the lumen patency to determine which surgical approach to use.^[23]^ Intraoperative angiography can determine the placement and size of the main stent. In patients with aortic dissection, the diameter of the aortic stent should be 5% to 10% larger than the inner diameter of the host vessel, which can avoid the occurrence of proximal type I internal fistula. According to the vascular morphology, choosing a stent with moderate flexibility and appropriate inner diameter can reduce the occurrence of “beak phenomenon.”^[24]^ At the same time, multi-angle angiography is needed during the operation to clarify the position and angle relationship between the aortic arch and the branch vessels, and further clarify the puncture site, angle, and depth. After successful puncture rupture, it is necessary to start with small balloon and gradually replace the large balloon to expand the puncture site,^[25]^ to reduce the damage to the vessel wall and surrounding tissues. Compared with the in situ fenestration technique, the branch part of the Castor single-branch stent can be rotated in multiple directions by up to 150 degrees while maintaining patency, which means that it can fully adapt to different positions of the LSA opening,^[26]^ and because it does not require incision and separation of the brachial artery, it can reduce surgical trauma, blood loss and operation time. At the same time, because there is no need to puncture membrane rupture and expand the puncture site, the blood supply recovery time of the left upper limb is shorter, the dosage of contrast agent is lower, and the contrast agent has less damage to the kidney.

If the collateral circulation is not compensated enough, it can lead to the occurrence of left upper limb ischemic symptoms, such as left upper limb weakness and limited activity. It has been reported that the incidence of left upper limb ischemic symptoms is as high as 39.0% to 46.2% when LSA is covered during TEVAR.^[27,28]^ In this study, both the Castor branched stent technique and the in situ fenestration technique demonstrated a high patency rate of the subclavian artery stent during the follow-up period, with the patency rate of the Castor branched stent technique being 33/34 and that of the in situ fenestration technique being 39/41, further confirming the clinical efficacy of Castor branched stent and in situ fenestration technique in the treatment of TBAD with insufficient proximal landing zone.

The occurrence of postoperative complications is an important factor affecting the health of patients. The aggravation of postoperative endoleak and stent displacement can lead to the further development of dissection, and even cause dissection rupture, leading to the death of patients due to massive bleeding.^[29]^ In this study, endoleak occurred during in situ fenestration in 7 patients, 3 of them were cured by intraoperative coil embolization. Two cases disappeared spontaneously during follow-up, and 2 cases underwent reoperation due to aggravation of endoleak (one of which had stent deformation due to severe endoleak). Four patients without endoleak were found to have endoleak due to stent migration or other unknown factors during follow-up. Endoleak occurred in 1 patient with Castor branch stent graft and resolved spontaneously during follow-up. Therefore, it is necessary to fully evaluate the severity of endoleak and the necessity of immediate treatment after the occurrence of endoleak immediately during the procedure. Severe endoleak needs immediate treatment, and mild endoleak may disappear spontaneously after the procedure. However, if the endoleak is aggravated during the follow-up, or even stent deformation or displacement occurs, it needs timely reoperation.

## 5. Limitation

This study has several limitations. Firstly, it is a single-center study with a small sample size, and some patients were lost to follow-up, which may introduce analysis bias. Additionally, the follow-up period is relatively short, necessitating further monitoring of long-term efficacy. Moreover, there is a lack of information regarding whether patients received the same or different anticoagulants or antiplatelet medications during the follow-up period, as well as whether they underwent similar or different treatments that may affect follow-up outcomes. This lack of data represents another limitation of the study design.

## 6. Conclusion

when Stanford type B aortic dissection involves LSA, either in situ fenestration technique or Castor branched stent graft can well preserve and reconstruct LSA, which not only extends the surgical indications of TEVAR, but also has the advantages of high success rate, few complications, and good short - and medium-term efficacy. Further comparative analysis showed that compared with the in situ single fenestration technique, we find that during surgery, patients in the Castor branched stent group experienced less blood loss, shorter operation time, shorter left upper limb blood supply recovery time, lower incidence of stent leakage, less contrast agent consumption, and higher technical success rate, which showed better clinical application potential.

## Acknowledgments

We appreciate the invaluable work done by the medical staff of the pathology department, including the nurses, and the radiologists.

## Author contributions

**Conceptualization:** Xiang Weng.

**Data curation:** Xiang Weng.

**Funding acquisition:** Weimin Zhou.

**Methodology:** Xiang Weng, Teng Zhang, YiLiang Hu, XianGui Li.

**Validation:** Xiang Weng.

**Visualization:** Xiang Weng.

**Writing – original draft:** Xiang Weng, Teng Zhang, YiLiang Hu, XianGui Li.

**Writing – review & editing:** Xiang Weng, Teng Zhang, YiLiang Hu, XianGui Li.
